# Gas fermentation for commodity chemicals and fuels

**DOI:** 10.1111/1751-7915.12763

**Published:** 2017-07-11

**Authors:** Frank R. Bengelsdorf, Peter Dürre

**Affiliations:** ^1^ Institut für Mikrobiologie und Biotechnologie Universität Ulm Albert‐Einstein‐Allee 11 89081 Ulm Germany

## Abstract

Gas fermentation is a microbial process that contributes to at least four of the sustainable development goals (SDGs) of the United Nations. The process converts waste and greenhouse gases into commodity chemicals and fuels. Thus, world's climate is positively affected. Briefly, we describe the background of the process, some biocatalytic strains, and legal implications.

Currently, two of the greatest challenges facing industry and society are the future sustainable production of chemicals and fuels from non‐food resources while at the same time reducing Greenhouse gas (GHG) emissions. The whole world needs to build a low‐carbon and climate‐resilient industrial environment by moving away from fossil fuels and investing in clean chemical and energy generation. Faced with this challenge, gas fermentation represents a disruptive technology that will bring transformational changes to industry and society (Fig. 1). Bacterial synthesis gas (syngas) fermentation is a microbial process that contributes to goals of the 2030 agenda for sustainable development (www.un.org/ga/search/view_doc.asp?symbol=A/RES/70/1&Lang=E). In this microbial process, GHG such as carbon monoxide (CO) and carbon dioxide (CO_2_) are fixed by a biocatalyst that simultaneously produces commodity chemicals or fuels. In 2015, the companies LanzaTech, ArcelorMittal and Primetals Technologies set up a corporation to build an industrial‐scale ethanol production facility in Ghent, Belgium (LanzaTech, 2015). The syngas fermentation technology has the potential to take urgent action to combat climate change and its impacts (goal 13) by reducing GHG emissions, if applied at industrial‐scale in several facilities with considerable amounts of exhaust waste gases. The use of this autotrophic acetogenic biocatalyst enables the sustainable consumption of CO and CO_2_ and provides sustainable production patterns of commodity chemicals or fuels (goal 12).

**Figure 1:**
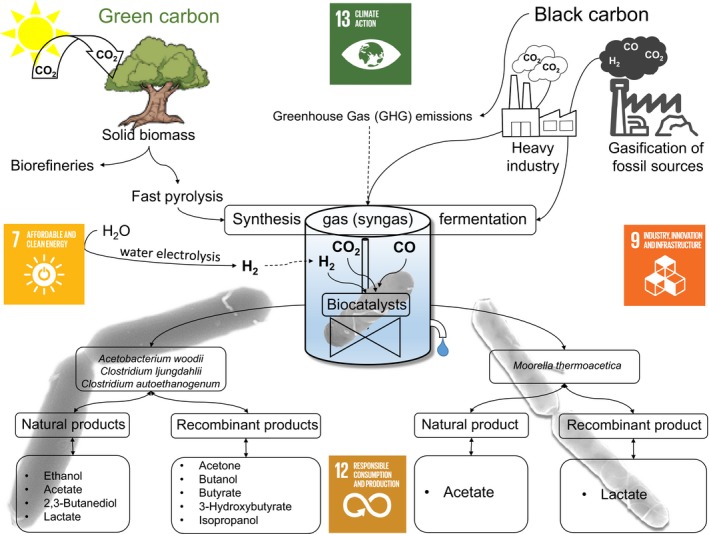
Graphical abstract of the gas fermentation process and links to sustainable development goals.

The syngas fermentation technology is expected to achieve a breakthrough once the industrial‐scale facility in Ghent is operating and producing 47 000 tons of ethanol per annum from waste gases originating from the steelmaking process. Within the next 10 years, this technology has the potential to build up a resilient infrastructure to capture GHG emissions from industrial waste gas streams, to promote sustainable industrialization by producing valued products and foster innovation in this field (goal 9).

Gas fermentation will also provide jobs and further career opportunities in academic and industrial bodies. Academia needs to train a cadre of researchers, able to apply their skills to the future societal challenges facing the world. Gas fermentation will also provide cutting edge technology to innovative enterprises all over the world. This might not be trivial, as competitors of LanzaTech, namely INEOS Bio and Coskata, already struggled on their route to establish an industrial‐scale biofuel production facility. However, it sets an ambitious goal which needs to be reached in order to maintain industrial and living standards on a planet with limited resources.

Gas fermentation also provides novel opportunities for renewable energy (goal 7). Currently, electricity prices face negative values, when too much wind or solar energy is fed into the grid. This surplus electricity could be used for hydrogen generation by electrolysis of water. Although generally considered to be an uneconomic process, it could help in such peak times to reduce carbon dioxide with help of hydrogen to produce industrially high‐value chemicals by acetogenic bacteria. Thus, surplus electricity from renewable resources can be used for production of platform chemicals with simultaneous reduction in GHG.

Here, we outline the background and recent developments in the field of bacterial syngas fermentation. Syngas can have multiple origins, e.g. (i) gasification of coal, petroleum, natural gas and peat coak; (ii) certain industrial waste gas streams; (iii) gasification of solid waste and (iv) pyrolysis/gasification of solid biomass. Legally, only syngas from solid biomass can be considered as ‘green carbon’. If fossil sources, or products made from fossil sources, were used to generate syngas, it has to be considered as ‘black carbon’. However, the amounts of syngas derived from fossil sources outnumber the amounts generated via pyrolysis or gasification of solid biomass (Dahmen *et al*., 2017). Especially, the gasification of fossil sources is a well‐established process and the world gasification industry is growing rapidly as indicated in the ‘Worldwide Syngas Database’ (http://www.gasification-syngas.org/resources/world-gasification-database/). The database provides information about plant locations, number and type of gasifiers, syngas capacity, feedstock and products. In 2015, global syngas output was 148 gigawatts thermal (GWth) and if all upcoming plans are realized, the worldwide syngas capacity will increase up to 300 GWth in 2020. The database does not consider ‘industrial waste gas’ or any other relevant energy rich waste gas output.

The used biocatalysts (also called acetogens) are anaerobic bacteria that employ the reductive acetyl‐CoA pathway to fix CO and/or CO_2_ and subsequently produce biofuels such as ethanol, butanol or hexanol as well as biocommodities such as acetate, lactate, butyrate, hexanoate, 2,3 butanediol and acetone using syngas as carbon and energy source. Wood–Ljungdahl pathway is a synonym of acetyl‐CoA pathway, and the respective biochemistry has been elucidated in a number of recent reviews (Drake *et al*., 2008; Ragsdale, 2008; Schuchmann and Müller, 2014). All acetogens produce acetic acid as metabolic end‐product because the production contributes significantly to the energy conversion processes of the cells. A further important energy conversion is realized by building up a sodium ion or proton gradient over the cell membrane that is finally used to drive enzymes called ATPases that generate adenosine triphosphate (ATP), which is the energy currency of life.

Some acetogens such as *Clostridium ljungdahlii*,* Clostridium autoethanogenum* or *Clostridium carboxidivorans* are of special interest, as they can produce valuable metabolic products as indicated above. *Clostridium ljungdahlii* and *C. autoethanogenum* are the best studied organisms with respect to possible applications in syngas fermentation processes. They share a special metabolic feature that enables them to convert the compulsorily produced acetate completely into the valuable product ethanol (Abubackar *et al*., 2016). Furthermore, both bacterial strains are closely related to each other and have the tremendous advantage that they are genetically accessible (Bengelsdorf *et al*., 2016). Thus, metabolic engineering of the bacterial cells is feasible and several recombinant strains have been constructed that produce biocommodities such as isopropanol, butyrate, butanol and 3‐hydroxybutyrate (Liew *et al*., 2016).

*Clostridium carboxidivorans* is of interest because it produces hexanol and hexanoic acid from syngas natively, which was shown simultaneously by two different groups (Phillips *et al*., 2015; Ramió‐Pujol *et al*., 2015). As this bacterium also produces butanol and ethanol, the term HBE (hexanol, butanol, ethanol) fermentation was introduced by Fernández‐Naveira *et al*. (2017). HBE fermentation is deduced from ABE (acetone, butanol, ethanol) fermentation that is known for solventogenic bacteria such *Clostridium acetobutylicum* and related bacteria (Bengelsdorf *et al*., 2017).

*Acetobacterium woodii* is a further acetogen of special interest, as it is used as model organism to study the metabolism of sodium‐dependent acetogenic bacteria in detail. The bacterium is also genetically accessible, and its metabolism was engineered to produce acetone (Hoffmeister *et al*., 2016). The available genetic tools offer several options to manipulate the metabolism of *A. woodii* cells and to learn more about their autotrophic lifestyle.

*Moorella thermoaceticum* is the acetogen that has been used to elucidate the biochemistry of the Wood–Ljungdahl pathway over decades of years. It grows under moderately thermophilic conditions (55 °C) and has also been genetically engineered to produce lactate (Kita *et al*., 2013; Iwasaki *et al*., 2017). A thermophilic acetogenic biocatalyst offers advantages for the syngas fermentation process. These include a reduced risk of contaminations, reduced costs for process cooling requirements and higher metabolic as well as diffusion rates.

Finally, a note on legislation, current regulations address the origin of carbon as the essential determinant for a product to be ‘bio’ or not. So, fuels made by microorganisms can be referred to as ‘biofuels’ only, when their substrate stems from biological material. Clearly, this does currently not apply to autotrophic acetogens when CO or CO_2_ are resulting from industrial processes (e.g. steel mills, chemical plants). It would, however, if CO_2_ is stemming from biomass gasification. And this is irrespective of the fact that in both cases microorganisms as biological catalysts are performing the conversion! This clearly will hamper companies, which are introducing the cutting edge technology of gas fermentation and are looking for financial benefits of qualifying under today's biofuels legislation, both in Europe and the USA. Thus, scientists need to emphasize that such misleading regulations should be corrected, in scientific publications as well as in information to politicians.

## Conflict of interest

None declared.

## References

[mbt212763-bib-0001] Abubackar, H.N. , Bengelsdorf, F.R. , Dürre, P. , Veiga, M.C. , and Kennes, C. (2016) Improved operating strategy for continuous fermentation of carbon monoxide to fuel‐ethanol by clostridia. Appl Energy 169: 210–217.

[mbt212763-bib-0002] Bengelsdorf, F.R. , Poehlein, A. , Linder, S. , Erz, C. , Hummel, T. , Hoffmeister, S. , *et al* (2016) Industrial acetogenic biocatalysts: a comparative metabolic and genomic analysis. Front Microbiol 7: 1036. 27458439 10.3389/fmicb.2016.01036 PMC4935695

[mbt212763-bib-0003] Bengelsdorf, F.R. , Poehlein, A. , Flitsch, S.K. , Linder, S. , Schiel‐Bengelsdorf, B. , Stegmann, B.A. , *et al*, (2017) Host Organisms: *Clostridium acetobutylicum*/*Clostridium beijerinckii* and related organisms In Industrial Biotechnology: Microorganisms. Wittmann C., Liao J.C. (eds). Weinheim, Germany: Wiley‐VCH Verlag GmbH & Co. KGaA.

[mbt212763-bib-0004] Dahmen, N. , Henrich, E. and Henrich, T. (2017) Synthesis gas biorefinery. In Advances in Biochemical Engineering/Biotechnology. Scheper, T., Belkin, S., Bley, T., Bohlmann, J., Gu, M.B., Hu, W.S. *et al*, (eds) p1–29. https://doi.org/10.1007/10_2016_63. 10.1007/10_2016_63 28331960

[mbt212763-bib-0005] Drake, H.L. , Gößner, A.S. , and Daniel, S.L. (2008) Old acetogens, new light. Ann N Y Acad Sci 1125: 100–128. 18378590 10.1196/annals.1419.016

[mbt212763-bib-0006] Fernández‐Naveira, Á. , Veiga, A.C. , and Kennes, C. (2017) H‐B‐E (Hexanol‐Butanol‐Ethanol) fermentation for the production of higher alcohols from syngas/waste gas. J Chem Technol Biotechnol 92: 712–731.

[mbt212763-bib-0007] Hoffmeister, S. , Gerdom, M. , Bengelsdorf, F.R. , Linder, S. , Flüchter, S. , Öztürk, H. , *et al* (2016) Acetone production with metabolically engineered strains of *Acetobacterium woodii* . Metabol Eng 36: 37–47. 10.1016/j.ymben.2016.03.001 26971669

[mbt212763-bib-0008] Iwasaki, Y. , Kita, A. , Yoshida, K. , Tajima, T. , Yano, S. , and Shou, T. (2017) Homolactic acid fermentation by the genetically engineered thermophilic homoacetogen *Moorella thermoacetica* ATCC 39073. Appl Environ Microbiol 83: e00247–17. 28159797 10.1128/AEM.00247-17 PMC5377493

[mbt212763-bib-0009] Kita, A. , Iwasaki, Y. , Sakai, S. , Okuto, S. , Takaoka, K. , Suzuki, T. , *et al* (2013) Development of genetic transformation and heterologous expression system in carboxydotrophic thermophilic acetogen *Moorella thermoacetica* . J Biosci Bioeng 115: 347–352. 23177215 10.1016/j.jbiosc.2012.10.013

[mbt212763-bib-0010] LanzaTech . (2015) Arcelor Mittal, LanzaTech and Primetals Technologies announce partnership to construct breakthrough €87m biofuel production facility. URL http://www.lanzatech.com/arcelormittal-lanzatech-primetals-technologies-announce-partnership-construct-breakthrough-e87m-biofuel-production-facility/ [accessed May 25, 2017]

[mbt212763-bib-0011] Liew, F. , Martin, M.E. , Tappel, R.C. , Heijstra, B.D. , Mihalcea, C. , and Köpke, M. (2016) Gas fermentation‐ a flexible platform for commercial scale production of low‐carbon‐fuels and chemicals from waste and renewable feedstocks. Front Microbiol 7: 694. 27242719 10.3389/fmicb.2016.00694 PMC4862988

[mbt212763-bib-0012] Phillips, J.R. , Atiyeh, H.K. , Tanner, R.S. , Torres, J.R. , Saxena, J. , Wilkins, M.R. , and Huhnke, R.L. (2015) Butanol and hexanol production in *Clostridium carboxidivorans* syngas fermentation: medium development and culture techniques. Bioresour Technol 190: 114–121. 25935391 10.1016/j.biortech.2015.04.043

[mbt212763-bib-0013] Ragsdale, S.W. (2008) Enzymology of the Wood‐Ljungdahl pathway of acetogenesis. Ann N Y Acad Sci 1125, 129–136. 18378591 10.1196/annals.1419.015 PMC3040112

[mbt212763-bib-0014] Ramió‐Pujol, S. , Ganigué, R. , Bañeras, L. , and Colprim, J. (2015) Incubation at 25 °C prevents acid crash and enhances alcohol production in *Clostridium carboxidivorans* P7. Bioresour Technol 192: 296–303. 26046429 10.1016/j.biortech.2015.05.077

[mbt212763-bib-0015] Schuchmann, K. , and Müller, V. (2014) Autotrophy at the thermodynamic limit of life: a model for energy conservation in acetogenic bacteria. Nat Rev Microbiol 12: 809–821. 25383604 10.1038/nrmicro3365

